# Impact of Ophthalmic Knowledge Assessment Program Scores and Surgical Volume on Subspecialty Fellowship Application in Ophthalmology Residency: Retrospective Cohort Study

**DOI:** 10.2196/60940

**Published:** 2024-11-13

**Authors:** Amanda Kay Hertel, Radwan S Ajlan

**Affiliations:** 1 University of Kansas School of Medicine, 7400 State Line Road, Prairie Village, KS, 66208-34444, United States, 1 913-588-6600, 1 913-588-0888

**Keywords:** residency, fellowship, ophthalmology, OKAP, surgical training, ophthalmology resident, ophthalmology residency program, examination, surgical volume exposure, fellowship training, surgical volume, exposure, Ophthalmic Knowledge Assessment Program

## Abstract

**Background:**

Ophthalmology residents take the Ophthalmic Knowledge Assessment Program (OKAP) exam annually, which provides percentile rank for multiple categories and the total score. In addition, ophthalmology residency training programs have multiple subspecialty rotations with defined minimum procedure requirements. However, residents’ surgical volumes vary, with some residents exceeding their peers in specific subspecialty rotations.

**Objective:**

This study aims to identify if there is a difference in OKAP examination scores and surgical volume exposure during ophthalmology residency training between nonfellowship and fellowship applicants and among various subspecialties.

**Methods:**

A retrospective review of OKAP scores and surgical procedure numbers of graduating residents in an accredited academic ophthalmology residency program in the Midwest United States was conducted. Data were collected from 2012 to 2022.

**Results:**

A total of 31 residents were identified. Most residents decided to pursue fellowship training upon graduation (20/31, 65% residents), and the rest chose to practice comprehensive ophthalmology (11/31, 35% residents). A total of 18/31 residents had OKAP score reports available. The fellowship group outperformed the nonfellowship group in multiple subsections and the total exam (*P*=.04). Those pursuing fellowship training in glaucoma performed higher on the Glaucoma section (*P*=.004) and the total exam (*P*=.005). Residents pursuing cornea performed higher on nearly all subsections, including External Disease and Cornea (*P*=.02) and the total exam (*P*=.007). The majority of the surgical volume exposure was identical between fellowship and nonfellowship groups. Those who pursued glaucoma fellowship performed more glaucoma filtering and shunting procedures (*P*=.03). Residents going into pediatrics fellowship were primary surgeons in more strabismus cases (*P*=.01), assisted in fewer strabismus cases (*P*<.001), and had no difference in the total number of strabismus surgeries.

**Conclusions:**

In our program, residents pursuing fellowship training had higher OKAP scores on multiple sections and the total exam. There was no significant difference in the overall surgical volume averages between fellowship and nonfellowship groups, but few differences existed in subspecialty procedures among fellowship applicants. Larger multicenter studies are needed to clarify the relationship between OKAP scores and ophthalmology fellowship decisions nationwide.

## Introduction

### Fellowship and Subspecialty Statistics

The number of ophthalmology residents who pursue further fellowship training has been increasing for more than a decade [1]. In 2005, around 64% of ophthalmology residents in the United States pursued subspecialty training, while 36% pursued comprehensive ophthalmology [2]. However, between 2012 and 2017, the percentage of ophthalmology residents in the United States pursuing subspecialty training increased to 70.3% [3]. Ophthalmology residency graduates in Canada have similar statistics, with 64% pursuing subspecialty training [4]. In the United States, vitreoretinal (36%), cornea (25%), glaucoma (13%), oculoplastic (10%), and pediatric (10%) fellowships are the most common [2]. Fewer ophthalmologists pursued fellowship training in anterior segment (2%), neuro-ophthalmology (0.7%), and uveitis (0.7%) [2]. These percentages are similar to the 2017 through 2022 San Francisco Match Data [5]. The match rate for all ophthalmology residents applying for fellowship is 73.7% [3]. Subspecialties with the greatest number of positions offered each year include retina, cornea, glaucoma, pediatrics, and uveitis [3]. The match rate was highest for retina, followed by cornea, glaucoma, pediatrics, and uveitis [3].

### Factors Influencing the Decision to Pursue a Fellowship

Based on a survey of ophthalmology residents, the top factors for deciding to pursue a fellowship include the desire for additional surgical training, additional clinical training, and increased job market competitiveness [6]. This is similar to a study stating that gaining more special skills and working with new technology were the top motivating factors for fellowship [7]. Another study found that acquiring special skills, perceived more favorable job market, and prestige were the top motivating factors for pursuing subspecialty training [2]. In contrast, those who pursued comprehensive ophthalmology were motivated by the anticipated work hours and geographical preference [2]. Other studies have also found that those going into comprehensive ophthalmology had significantly higher student loan amounts [7]. It is also noted that those deciding to do subspecialty training were more likely to intend to practice in an academic setting [2,7]. In addition, ophthalmology residents applying for fellowship had significantly more first-author publications than those going into comprehensive ophthalmology [7].

Studies have found that gender has no significant impact on the decision to pursue subspecialty training following ophthalmology residency [2,4,6,8]. However, there were gender differences among specific subspecialties. It was found that significantly more males pursued vitreoretinal surgery, while more females pursued strabismus and pediatric ophthalmology [4,8]. It has also been found that more males pursued oculoplastic [4] and anterior segment [8] fellowships. Females were also more likely to pursue neuro-ophthalmology [8]. Other studies have shown that age, ethnicity, marital status, presence of children, or level of educational debt had no statistically significant impact on the decision to pursue subspecialty training [2].

A survey done in 2005 on graduating residents found that the number of ocular procedures performed during residency did not significantly differ between residents who decided to do a fellowship and those who went into comprehensive ophthalmology [2]. However, there may be more variation when looking at specific subspecialties, as this study only evaluated fellowship versus no fellowship cohorts. Other factors, including elective time, career counseling, and the amount of dedicated time for research, also did not impact the decision to pursue a fellowship [2].

### Factors Influencing the Decision to Pursue Specific Subspecialties

One study evaluated residents’ decisions to pursue a fellowship in neuro-ophthalmology [9]. The top reasons graduating residents decided not to pursue this fellowship included stronger interests in other fields, types of patients seen, no intraocular surgery, and the assumption that it is a nonsurgical discipline. Factors influencing the decision to pursue neuro-ophthalmology included interest in clinical diseases and interaction with other specialty fields. There were no differences between the groups regarding the degree of exposure to neuro-ophthalmology in medical school, the presence of a dedicated neuro-ophthalmology rotation in residency, or the rotation timing [9].

Another study evaluating the decisions impacting glaucoma fellowship found that residents entering a glaucoma fellowship had performed more glaucoma filtering procedures, were less likely to publish a paper, and were less likely to have time allocated for research than residents who pursued different fellowship training. Those seeking glaucoma fellowship also found challenging diagnostic problems, types of patient problems, academic careers, and working with new technology as less important. Residents pursuing glaucoma fellowship also decided later than residents selecting other subspecialties [10].

A recent study published in 2023 evaluated ophthalmology residents’ perceptions of pediatric ophthalmology, and it was found that the desire to work with children was the most significant factor in pursuing a pediatric ophthalmology residency. The majority of residents also did not believe pediatric ophthalmology to be a prestigious specialty [11], and some residents also found pediatric patients difficult to examine [12], both deterrents to pursuing pediatric ophthalmology. In addition, concerns of economic factors and compensation in this field have also been discussed [11-13].

### Ophthalmic Knowledge Assessment Program

The Ophthalmic Knowledge Assessment Program (OKAP) is a multiple-choice nationwide examination used to assess the ophthalmic knowledge of ophthalmology residents compared with their peers at the same training stage (postgraduate years [PGY] 2, 3, and 4). OKAP exams are taken electronically in a supervised environment in March each year. Residents in their first postgraduate year do not take the OKAP exam because the first year is spent rotating through other specialties as an internship year with little time in ophthalmology. OKAP exam scores are frequently requested when applying for competitive fellowship programs. Essential factors to score well on this examination include increased time spent studying, the use of question banks, and the incorporation of OKAP materials into the residency program [14]. In a study surveying retina, glaucoma, and cornea fellowship programs, OKAP scores were ranked as moderately important by program directors for fellowship applications [15]. It is notable that not all fellowship programs evaluate OKAP scores and that OKAP scores are only one indicator of proficiency in ophthalmology. Despite the first national OKAP exam occurring in 1968 [16], no studies have evaluated the performance of OKAP scores on the decision to pursue fellowship training after an ophthalmology residency.

### Significance

There is limited literature examining the factors that influence fellowship training selection, especially for specific subspecialties. No studies have evaluated the impact of OKAP scores and intraocular procedural volume on specific subspecialty of choice by residents. This study aims to better understand the factors leading to selecting a subspecialty after graduating from an ophthalmology residency program by evaluating the OKAP scores and the number of surgical procedures from graduating residents. This research can then provide valuable information to ophthalmology residency program directors as programs can be better designed to graduate ophthalmologists in subspecialties of need.

## Methods

### Study Methods

A retrospective review of OKAP scores and intraocular procedure numbers of graduating residents in an accredited ophthalmology residency program in the Midwest United States was conducted. Data were collected for all ophthalmology residents graduating from the program between 2012 to 2022. Data collection included OKAP scores, gender, fellowship decision, and procedural volume for all prior residents meeting the inclusion criteria (graduation date from 2012 to 2022). OKAP score reports were available on the San Francisco Match website for residents graduating between 2017 and 2022. OKAP examinations are required and taken by ophthalmology residents during their second, third, and fourth residency training years. The exam is currently taken online while proctored in person. All OKAP scores were available, but only OKAP scores from each residents’ final program year were used in data analysis. This is because the final year OKAP scores are the most representative of cumulative ophthalmic knowledge, and are closer to the final decision of practice or fellowship.

### Statistical Analysis

Descriptive statistics were completed for the demographics, number of surgical cases, and OKAP scores among the various groups of interest. For comparing the various groups, *t* tests were used with the settings of two-sample unequal variance with a one-tailed distribution. Statistical analysis was performed using Microsoft Excel.

### Ethical Considerations

Institutional review board (IRB) approval was obtained from the University of Kansas Medical Center (study ID: 00150405). This was a retrospective review with secondary analysis of data. The IRB approval covers secondary analysis performed without the need for additional consent. All data were deidentified immediately following initial collection. No compensation was provided.

## Results

### Overview

A total of 31 residents were identified. Most residents decided to pursue fellowship training upon graduation (20/31, 65% residents), and the rest decided to practice comprehensive ophthalmology (11/31, 35%residents). Overall, 20 separate fellowships were completed by residents in Glaucoma (7/20, 35% fellowships), Surgical Retina (5/20, 25% fellowships) and Medical Retina (2/20, 10% fellowships), Cornea (4/20, 20% fellowships), and Pediatrics (2/20, 10% fellowships). No residents completed fellowships in Oculoplastics, Uveitis, or Anterior Segment in this residency program.

Out of the 31 residents identified, 42% (13/31) were female, while 58% (18/31) were male (Table 1). Of those who pursued fellowship (n=20), 50% (10/20) were female, and 50% (10/20) were male, while those who did not pursue a fellowship were 27% (3/11) female and 73% (8/11) male. When looking at specific subspecialties, 29% (2/7) of residents who pursued a Glaucoma fellowship were female. In addition, 50% (2/4) of Cornea fellowships, 100% Pediatric fellowships (2/2), 0% (0/2) of Medical Retina fellowships, and 60% (3/5) of Surgical Retina fellowships were female.

**Table 1.** Resident demographics (N=31).

Demographics	Female residents, n/N (%)	Male residents, n/N (%)
Total	13/31 (42)	18/31 (58)
Pursued fellowship	10/20 (50)	10/20 (50)
No fellowship; comprehensive ophthalmology	3/11 (27)	8/11 (73)
Glaucoma	2/7 (29)	5/7 (71)
Cornea	2/4 (50)	2/4 (50)
Pediatrics	2/2 (100)	0/2 (0)
Medical Retina	0/2 (0)	2/2 (100)
Surgical Retina	3/5 (60)	2/5 (40)

### Fellowship Versus No Fellowship

A total of 18 OKAP score reports since 2017 were available. Around half of these residents pursued a fellowship (56%, 10/18 residents), while the others pursued comprehensive ophthalmology (44%, 8/18 residents). On the OKAP scores, the fellowship group outperformed the nonfellowship group in General Medicine (*P*=.03), Ophthalmic Pathology and Intraocular Tumors (*P*=.04), Lens and Cataract (*P*=.04), and Total Exam (*P*=.04). The difference in OKAP percentile rank compared with the entire group average for the fellowship and no fellowship cohorts is detailed in Table 2.

**Table 2.** Difference in Ophthalmic Knowledge Assessment Program score average percentile rank compared with the whole cohort.

	General medicine	Fundamentals and principles of ophthalmology	Clinical optics	Ophthalmic pathology and intraocular tumors	Neuro-ophthalmology	Pediatric ophthalmology and strabismus	Orbit, eyelids, and lacrimal system	External disease and cornea	Intraocular inflammation and uveitis	Glaucoma	Lens and cataract	Retina and vitreous	Refractive surgery	Total exam
Fellowship (n=10)	13^a^	12	8	10^a^	−1	10	4	8	10	9	13^a^	8	6	12^a^
No fellowship (n=8)	−17^a^	−14	−10	−13^a^	1	−13	−5	−10	−13	−11	−16^a^	−10	−8	−15^a^
Glaucoma (n=6)	12	10	9	19^b^	9	13	16^a^	8	13	22^b^	9	8	8	21^b^
Cornea (n=2)	18^a^	17^a^	23^b^	19	1	27^b^	23^b^	14^a^	35^b^	24^b^	33^b^	11	35^b^	21^b^
Medical Retina (n=2)	11	12	−7	−24	−32^a^	−15^a^	−51^a^	2	−24	−47^b^	4	6	−26^a^	−23

a *P*<.05*.*

b *P*<.01

All residents had surgical case number reports available. There were minimal differences between the fellowship and no fellowship group in procedural volume. However, the fellowship cohort did assist in more oculoplastic and orbit cases (*P*=.02) and more eyelid laceration cases (*P*=.02). All other surgical volume categories had no statistical significance between the fellowship and no fellowship groups (Tables S1-S4 in Multimedia Appendix 1).

### Subspecialty

The specialties represented among the OKAP score reports were Glaucoma (60%, 6/10 residents), Cornea (20%, 2/10 residents), and Medical Retina (20%, 2/10 residents). Analysis of OKAP scores was then performed on the subspecialty cohorts compared with all other residents. Those who ended up pursuing fellowship training in glaucoma performed higher on Ophthalmic Pathology and Intraocular Tumors (*P*=.004), Orbit, Eyelids, and Lacrimal System (*P*=.02), Glaucoma (*P*=.004), and on total exam (*P*=.005). Those pursuing cornea performed higher on nearly all subsections and ontotal exam (*P*=.007). The sections they scored higher on include General Medicine (*P*=.01), Fundamentals and Principles of Ophthalmology (*P*=.02), Clinical Optics (*P*=.002), Pediatric Ophthalmology and Strabismus (*P*<.001), Orbit, Eyelids, and Lacrimal System (*P*=.004), External Disease and Cornea (*P*=.02), Intraocular Inflammation and Uveitis (*P*<.001), Glaucoma (*P*=.004), Lens and Cataract (*P*=.002), and Refractive Surgery (*P*<.001). The other fellowship represented in the OKAP scores was Medical Retina. Those who ended up pursuing medical retina did perform lower on some subsections. However, their total exam score and their Retina and Vitreous section scores were no different than the rest of the graduates. Residents who ended up pursuing medical retina scored lower on Neuro-ophthalmology (*P*=.05), Pediatric Ophthalmology and Strabismus (*P*=.02), Orbit, Eyelids, and Lacrimal System (*P*=.02), Glaucoma (*P*<.001), and Refractive Surgery (*P*=.02).

The procedural volume completed by the various subspecialties was also analyzed. Those who pursued a fellowship in glaucoma performed fewer cases of panretinal laser photocoagulation (*P*=.01) and strabismus (*P*=.04) as primary. They, however, assisted on more eyelid laceration cases (*P*=.03) and had more total glaucoma filtering and shunting procedures (*P*=.03). Those seeking cornea had fewer assist cases for laser trabeculoplasties (*P*=.004), laser iridotomies (*P*=.01), and chalazion excisions (*P*=.002). The cornea fellowship group assisted more and had a higher total number of retinal vitreous cases (*P*=.002 and .007, respectively). They also performed more open globe trauma cases (*P*=.03). Residents who went into pediatric ophthalmology performed fewer intravitreal injections (*P*<.001), and eyelid laceration repair (*P*=.002), as primary. They also had fewer total keratorefractive surgeries (*P*=.003) and globe trauma cases (*P*=.009). However, those pursuing pediatrics were the primary surgeons for more strabismus cases (*P*=.01) and assisted in fewer strabismus cases (*P*<.001). There was no significant difference in the total number of strabismus surgeries performed by graduates who pursued pPediatric Ophthalmology compared with other residents. Those going into Medical Retina had fewer total keratoplasty cases (*P*=.007) and assisted in more ptosis and blepharoplasty cases (*P*=.01). Those going into surgical retina had fewer total case numbers for cataract, Yttrium Aluminum Garnet capsulotomy, laser trabeculoplasty, and glaucoma surgeries (*P*=.02, .002, <.001, <.001, respectively). The surgical retina group also had fewer total pterygium or conjunctival cases (*P*=.04) and keratorefractive surgeries (*P*=.001; Tables S1-S4 in Multimedia Appendix 1).

## Discussion

### Principal Findings

To the best of our knowledge, this is the first study to evaluate the effect of OKAP scores on fellowship choice. In this study, it was found that most graduates from this residency program pursued fellowship training, with the most common fellowships being Glaucoma, Surgical Retina, Cornea, Medical Retina, and Pediatrics, respectively. When analyzing the OKAP scores, it was found that residents pursuing fellowship training scored higher on the total OKAP exam and on multiple subsections. It was also found that residents applying for specific subspecialties scored higher on that subsection of the OKAP examination. In addition, the procedural volume did not significantly differ between the fellowship and nonfellowship groups with some variations when analyzing specific subspecialties.

### OKAP Scores Difference Between Fellowship and Nonfellowship

In multiple categories and on total exam, ophthalmology residents who pursued fellowship training scored higher than those who did not. One possible explanation for this difference is that OKAP scores are sometimes included in a Fellowship Application and have been identified as a moderately important aspect of a fellowship application by some program directors [15]. Residents considering a fellowship may have prepared more for the exam to be competitive for fellowship applications. In addition, this finding could suggest that residents who scored high on the OKAP were later influenced to pursue fellowship training due to their more competitive applications. Since this is a retrospective review, it is unknown whether residents entered this program with a preexisting interest in fellowship. Therefore, higher OKAP scores in general may serve as a predictor for residency programs that a resident is more likely to pursue fellowship training.

### Ophthalmic Knowledge Assessment Program Score Differences Among Subspecialties

Those who pursued glaucoma fellowship also scored higher on total exam and in multiple subsections, including Glaucoma, compared with the rest of the cohort. In addition, those who pursued cornea fellowship outperformed the fellowship and nonfellowship groups on total exam and in many subcategories, including External Disease and Cornea. This could indicate that residents who decided to apply for a specific fellowship in a certain specialty had increased interest and knowledge in the field, spent more time learning that content, and scored higher on the associated OKAP sections. This could be one way for residency programs to identify applicants more likely to pursue fellowship training in a specific area. Those pursuing medical retina scored lower on some OKAP subsections but not on total exam or the Retina and Vitreous section. One potential reason is that medical retina (and retina in general) has an overall higher match rate, and a greater number of positions offered, making it less competitive than some of the other specialties [3]. This may mean OKAP scores were perceived to have less impact on matching into medical retina programs. It is also noted that while there are variations in the OKAP scores, all residents have successfully graduated from the residency program and are board-certified.

### Procedural Volume Between Fellowship and Nonfellowship

Interestingly, in our study, when comparing the procedural volume of residents who pursued fellowship to those who did not, the only statistically significant difference was in the oculoplastics and orbit and eyelid laceration categories, despite no residents seeking fellowship in oculoplastics. This agrees with other studies that have shown the number of ocular procedures did not significantly differ between residents going into fellowship programs versus comprehensive ophthalmology [2]. It is also noted that this study evaluates a greater variety of procedures due to various technological advancements since 2005. This shows that while students might have different career paths, the bulk of surgical training during ophthalmology residency is the same. This is also important for those going into comprehensive ophthalmology, as comprehensive ophthalmologists must be well-versed in many areas.

### Procedural Volume Differences Among Subspecialties

In this study, specific subspecialties had some variations within procedural volumes. For example, those who pursued glaucoma fellowship had a greater total number of glaucoma filtering and shunting procedures. This is consistent with another study that found residents entering a glaucoma fellowship performed more glaucoma filtering procedures [10]. This may mean those residents sought more opportunities to get involved in glaucoma cases and pursued extra training. Residents who ended up in pediatrics were the surgeons for more strabismus cases but had no statistically significant difference in the total number of strabismus cases. Interestingly, residents who went into cornea had no differences in corneal surgery procedural volume, and those who went into medical or surgical retina had no differences in retinal vitreous and intraretinal injections. It is also noted that despite these variations, all residents met the minimum training requirements in each area.

### Residency Demographics and Nonpursued Subspecialties

The percentage of program graduates deciding to pursue fellowship training (64.5%) is close to the approximately 70% of ophthalmology residency graduates in the United States who pursued fellowship training [3]. The lower percentage pursuing a fellowship in this specific program may be because this program has no associated fellowship programs. Those who selected this residency program may have been less motivated by fellowship programs initially. While retina, cornea, and pediatric percentages are similar to averages in the United States, significantly more residents from this program pursued glaucoma fellowship [2]. It is also noted that no residents pursued a fellowship in oculoplastics despite it being the fourth most common ophthalmology fellowship nationwide [2]. One potential explanation is that the Oculoplastics application is during PGY-3 year instead of PGY-4 like the others, meaning residents must make this decision early [17]. This is also considered to be one of the more competitive fellowship programs, which may deter residents from applying. In addition, no residents pursued a fellowship in uveitis, neuro-ophthalmology, or anterior segment. However, in the United States, these fellowship programs account for smaller percentages out of all fellowships [2].

### Gender

While the majority of residents in this study identified as male, half of the cohort who pursued fellowship training identified as female. Conversely, most residents who did not pursue fellowship training were male. This is consistent with other studies that have shown gender to have no significant impact on the decision to attain subspecialty training in ophthalmology [2,4,6,8]. At least 50% of residents in this program who pursued fellowships in pediatrics (100%, 2/2 residents), surgical retina (60%, 3/5 residents), and cornea (50%, 2/4 residents) identified as female. This is consistent with other studies that have found more female ophthalmologists complete fellowships in Pediatrics and Strabismus [4,8]. However, these studies also found most residents who pursued Surgical Retina fellowship identify as male [4,8]. Interestingly, in our cohort, the majority of residents who pursued Retina Surgery were female. This could be due to the small cohort size or program-specific variations like the presence of female surgical retina attendings.

### Other Factors Affecting Decision to Pursue a Fellowship

This study assessed the impact of OKAP scores and procedural volume on fellowship decision. Other studies and surveys have found that other important factors in the decision to subspecialize in ophthalmology include wanting additional training (both clinical and surgical) [2,6], acquiring specialized skills [7], working with new technology [7], increased job market competitiveness [2,6], and prestige [2]. In addition, those who have fellowship training are more likely to practice in academic settings [2,7], and to have first-author publications [7]. Therefore, residents who want to work in research or academics may be more inclined to subspecialize.

### Limitations

The limitations of this study include the small sample size of a single mid-western program. The limited sample size can limit the generalizability of this study in a larger program. In addition, this residency program did not have associated fellowship programs during the study period, which, may have influenced residents’ decision to pursue fellowship training, or attracted residents who wanted to maximize their surgical experience without fellows to practice comprehensive ophthalmology after graduation. Another limitation is the retrospective nature of this study, which does not allow examining if residents decided on fellowship before or after OKAP examination scores. It is unknown at what point in training each resident decided to pursue a fellowship. In addition, no residents decided to pursue training in certain fellowships (eg, oculoplastics and neuro-ophthalmology), so there are no data on those specific specialties.

### Strengths

This study was conducted in an ophthalmology residency program accredited by the Accreditation Council for Graduate Medical Education. All residents in the program passed their minimal required surgical procedure volumes. All graduates of this program are certified by the American Board of Ophthalmology or preparing to set the board exams if graduated in 2022 at the time of the paper submission.

### Conclusion

OKAP performance showed there were differences between fellowship and nonfellowship graduates in our program. Overall, residents in this program who pursued a fellowship scored higher than those who did not pursue a fellowship on multiple sections and on total OKAP examination. There were no significant differences in the overall surgical volume averages between fellowship and nonfellowship groups, but a few differences existed in subspecialty procedures among fellowship applicants. Despite these variations, all residents exceeded the minimum training requirements. Larger multicenter studies are needed to better clarify OKAP score relation to fellowship and subspecialty application decisions nationwide.

## Supplementary material

10.2196/60940 Multimedia Appendix 1 Supplementary data.

## Abbreviations

IRB: institutional review board
OKAP: Ophthalmic Knowledge Assessment Program
