# Supplementary material for: Impact of Ophthalmic Knowledge Assessment Program Scores and Surgical Volume on Subspecialty Fellowship Application in Ophthalmology Residency: Retrospective Cohort Study
Source: JMIR Med Educ. 2024 Nov 13;10:e60940. doi: 10.2196/60940 (PMC11611791; doi:10.2196/60940)
Supplement: Multimedia Appendix 1 [file mededu-v10-e60940-s001.docx]

**Table S1.** Average number of procedures completed during residency in Cataract, YAG Capsulotomy, Laser Trabeculoplasty, Laser Iridotomy, and Panretinal Laser Photocoagulation.

|  | Cataract | | | Laser Surgery - YAG Capsulotomy | | | Laser Surgery - Laser Trabeculoplasty | | | Laser Surgery - Laser Iridotomy | | | Laser Surgery - Panretinal Laser Photocoagulation | | |
| --- | --- | --- | --- | --- | --- | --- | --- | --- | --- | --- | --- | --- | --- | --- | --- |
|  | Surgeon | Assistant | Total | Surgeon | Assistant | Total | Surgeon | Assistant | Total | Surgeon | Assistant | Total | Surgeon | Assistant | Total |
| Fellowship (*n* = 20) | 207 | 134 | 340 | 29 | 2 | 30 | 11 | 2 | 12 | 9 | 1 | 10 | 23 | 2 | 26 |
| No Fellowship (*n* = 11) | 230 | 113 | 343 | 29 | 4 | 32 | 13 | 1 | 14 | 7 | 0 | 8 | 18 | 3 | 22 |
| Glaucoma (*n* = 7) | 221 | 163 | 384 | 32 | 2 | 33 | 13 | 3 | 16 | 8 | 1 | 10 | 15* | 3 | 19* |
| Cornea (*n* = 4) | 242 | 148 | 390 | 37 | 2 | 39 | 11 | 0** | 11 | 9 | 0* | 9 | 24 | 3 | 26 |
| Pediatrics (*n* = 2) | 194 | 128 | 321 | 27 | 0** | 27 | 10 | 1 | 10 | 13 | 1 | 14 | 26* | 0** | 26 |
| Medical Retina (*n* = 2) | 204 | 121 | 324 | 28 | 7 | 34 | 18 | 3 | 21 | 10 | 1 | 10 | 27 | 4 | 30 |
| Surgical Retina (*n* = 5) | 153* | 83* | 254* | 18* | 1** | 19** | 5** | 0* | 5** | 8 | 0* | 8 | 33 | 2 | 34 |

**p* < 0.05, ***p* < 0.01

**Table S2.** Average number of procedures completed during residency in Keratoplasty, Pterygium/Conjunctival and other Cornea, Corneal Surgery, Keratorefractive Surgery, and Strabismus.

|  | Keratoplasty | | | Pterygium/Conjunctival and other cornea | | | Corneal Surgery (keratoplasty, pterygium/conjunctival, other) | | | Keratorefractive Surgery | | | Strabismus | | |
| --- | --- | --- | --- | --- | --- | --- | --- | --- | --- | --- | --- | --- | --- | --- | --- |
|  | Surgeon | Assistant | Total | Surgeon | Assistant | Total | Surgeon | Assistant | Total | Surgeon | Assistant | Total | Surgeon | Assistant | Total |
| Fellowship (*n* = 21) | 3 | 12 | 15 | 8 | 8 | 16 | 11 | 20 | 31 | 1 | 13 | 14 | 24 | 21 | 44 |
| No Fellowship (*n* = 12) | 2 | 9 | 11 | 8 | 8 | 16 | 10 | 18 | 28 | 1 | 9 | 10 | 23 | 20 | 44 |
| Glaucoma (*n* = 9) | 3 | 13 | 16 | 11 | 8 | 19 | 13 | 20 | 33 | 1 | 13 | 14 | 19* | 23 | 43 |
| Cornea (*n* = 4) | 7 | 11 | 18 | 9 | 10 | 18 | 16 | 21 | 36 | 1 | 24 | 25 | 28 | 20 | 48 |
| Pediatrics (*n* = 2) | 1 | 13 | 14 | 4 | 9 | 13 | 5 | 22 | 27 | 1 | 6* | 7** | 31* | 11** | 41 |
| Medical Retina (*n* = 2) | 1 | 10 | 11** | 6 | 8 | 13 | 7 | 18 | 24 | 0** | 21 | 21 | 16 | 27 | 43 |
| Surgical Retina (*n* = 5) | 1* | 14 | 14 | 6 | 6 | 12* | 7* | 20 | 26 | 1 | 3** | 4** | 28 | 19 | 43 |

**p* < 0.05, ***p* < 0.01

**Table S3.** Average number of procedures completed during residency in Glaucoma, Retinal Vitreous, and Intravitreal Injection.

|  | Glaucoma – Filtering/Shunting Procedures | | | Retinal Vitreous | | | Intravitreal Injection | | |
| --- | --- | --- | --- | --- | --- | --- | --- | --- | --- |
|  | Surgeon | Assistant | Total | Surgeon | Assistant | Total | Surgeon | Assistant | Total |
| Fellowship (*n* = 21) | 13 | 10 | 22 | 6 | 39 | 45 | 57 | 2 | 58 |
| No Fellowship (*n* = 12) | 12 | 12 | 23 | 8 | 31 | 39 | 54 | 2 | 56 |
| Glaucoma (*n* = 9) | 13 | 14 | 27* | 4 | 34 | 38 | 49 | 2 | 51 |
| Cornea (*n* = 4) | 18 | 11 | 29 | 7 | 51** | 57** | 82 | 1 | 83 |
| Pediatrics (*n* = 2) | 14 | 5 | 19 | 4* | 38 | 41 | 23** | 2 | 24** |
| Medical Retina (*n* = 2) | 10 | 10 | 20 | 4 | 32 | 36 | 65 | 8 | 72 |
| Surgical Retina (*n* = 5) | 8* | 4** | 12** | 11 | 40 | 51 | 56 | 1 | 55 |

**p* < 0.05, ***p* < 0.01

**Table S4.** Average number of procedures completed during residency in Oculoplastic and Orbit, Eyelid Laceration, Chalazion Excision, Ptosis and Blepharoplasty, and Globe Trauma.

|  | Oculoplastic and Orbit | | | Oculoplastic and Orbit – Eyelid Laceration | | | Oculoplastic and Orbit – Chalazion Excision | | | Oculoplastic and Orbit – Ptosis/Blepharoplasty | | | Globe Trauma | | |
| --- | --- | --- | --- | --- | --- | --- | --- | --- | --- | --- | --- | --- | --- | --- | --- |
|  | Surgeon | Assistant | Total | Surgeon | Assistant | Total | Surgeon | Assistant | Total | Surgeon | Assistant | Total | Surgeon | Assistant | Total |
| Fellowship (*n* = 21) | 79 | 114* | 193 | 10 | 10* | 19 | 7 | 1 | 9 | 24 | 31 | 55 | 10 | 3 | 13 |
| No Fellowship (*n* = 12) | 85 | 80* | 165 | 12 | 5* | 17 | 7 | 2 | 8 | 21 | 24 | 45 | 11 | 3 | 14 |
| Glaucoma (*n* = 9) | 83 | 133 | 216 | 9 | 14* | 22 | 8 | 2 | 10 | 25 | 35 | 60 | 9 | 3 | 12 |
| Cornea (*n* = 4) | 77 | 100 | 177 | 13 | 5 | 18 | 8 | 0** | 8 | 19 | 31 | 50 | 13* | 3 | 16 |
| Pediatrics (*n* = 2) | 65 | 89 | 153 | 7** | 11 | 18 | 11 | 1 | 12 | 11 | 21 | 32 | 8 | 1* | 8** |
| Medical Retina (*n* = 2) | 73 | 107 | 179 | 11 | 9 | 20 | 6 | 2 | 7 | 33 | 37* | 70 | 10 | 2 | 12 |
| Surgical Retina (*n* = 5) | 86 | 109 | 195 | 11 | 8 | 17 | 5** | 1 | 8 | 27 | 27 | 53 | 12 | 4 | 14 |

**p* < 0.05, ***p* < 0.01
